# Feasibility of Oral Rabies Vaccination of Dogs in Mexico

**DOI:** 10.3390/tropicalmed10090244

**Published:** 2025-08-28

**Authors:** Verónica Gutiérrez Cedillo, Luis Antonio Montoya Mondragón, Jose Ramón Fernández Colín, Katharina Bobe, Ad Vos, Luis Armando Lecuona Olivares, Ruy López Ridaura

**Affiliations:** 1 CENAPRECE, Ministry of Health, Marina Nacional N°60, Piso 1 Col. Tacuba Miguel Hidalgo, Mexico City CP 11400, Mexico; ramon.fernandez@salud.gob.mx (J.R.F.C.); ruy.lopez@salud.gob.mx (R.L.R.); 2 Ministry of Health of the Queretaro State, Privada Circunvalación N°6, Col. Jardines de Queretaro, Querétaro CP 76020, Mexico; montoya-luis@hotmail.com; 3 Ceva Innovation Center GmbH, Am Pharmapark, 06861 Dessau-Rosslau, Germany; katharina.bobe@ceva.com (K.B.); ad.vos@ceva.com (A.V.); 4 United States Department of Agriculture, Sierra Nevada 115, Lomas de Chapultepec, Miguel Hidalgo, Mexico City CP 11000, Mexico; luis.lecuona@usda.gov

**Keywords:** rabies, oral vaccination, dogs, bait acceptance, immunogenicity, SPBN GASGAS

## Abstract

Mexico has not only successfully eliminated dog-mediated human rabies in recent years, but also the last rabies case in a dog infected with the canine variant of the rabies virus was reported in 2016. Mass dog vaccination campaigns were the cornerstone of these achievements. Unfortunately, the rabies virus still circulates in wildlife and, thus, spill-over infections in humans, livestock, and pets, including dogs, still occurs. Especially dogs that cohabit at interfaces shared with wildlife, like shepherd dogs, are at risk. These dogs are often free-roaming and difficult to restrain for vaccination purposes. Oral rabies vaccination (ORV) as an alternative vaccination strategy was tested in several rural villages in Querétaro State, Mexico. Bait acceptance and immunogenicity studies were conducted to test a licensed vaccine bait in terms of attractiveness and if the oral rabies vaccine strain, SPBN GASGAS, was able to induce an adequate immune response in local dogs, respectively. Although the egg(-flavored) bait was less well accepted (68.4%) by the dogs than the two other bait types included in the study, a bait made from boiled intestine segments (71.2%) and a bait with fish meal as an attractant (72.3%), dogs offered the egg bait were more often considered successfully vaccinated. 83.3% of the dogs offered an egg bait seroconverted during the immunogenicity study. Hence, ORV can be a suitable alternative by increasing the overall vaccination coverage of dogs that cannot be easily restrained and handled for vaccination.

## 1. Introduction

Mexico was the first country that met the validation requirements of the World Health Organization and was subsequently recognized to be free of dog-mediated human rabies in November 2019 [1,2]. Since 2006, no dog-mediated human rabies case has been reported in the country [3,4]. The last case of the canine variant in a dog was reported in 2016 [2]. This was achieved, among others, through mass dog vaccination (MDV) as part of the national rabies program starting in 1990, with about seven million dogs vaccinated [5]. The number of dogs vaccinated increased during subsequent years and reached a peak in 2014 when 17 million dogs were vaccinated [6]. Although the canine variant has disappeared from the dog population, the rabies virus (RABV) still circulates in wildlife and can be transmitted to dogs. For example, three cases in dogs were reported in 2017; in two dogs the virus isolated originated from vampire bats, and the third dog was infected with a skunk variant. Another, more recent case in 2020, concerned a puppy attacked by a skunk [4]. Hence, there is a risk that after such a spillover infection from wildlife to dogs, the virus can re-establish itself once more in the dog population. To circumvent such a scenario, it has been suggested to maintain the mass dog vaccination efforts from the past [4]. However, these campaigns are very time—and labor—intensive and thus costly. It may be difficult to obtain sustained political and financial support for such a program in the future, as few or no cases in dogs are observed. Alternative, more cost-effective strategies need to be investigated. For example, increasing the number of dogs vaccinated by the private sector; presently only 4–5 million of the estimated 44 million dogs are vaccinated by private veterinarians in Mexico [2]. Unfortunately, especially in rural areas, this transition cannot easily be implemented, if possible at all. Here, many dogs, like shepherd dogs, are free-roaming and cannot be restrained for parenteral vaccination but have a high risk of encountering wildlife. Oral rabies vaccination (ORV) could be a suitable method of reaching these dogs. With regained interest in ORV of dogs, field studies testing the feasibility of this tool have been conducted in many countries [7,8,9,10,11,12,13,14,15,16,17,18,19,20]. To investigate the feasibility of ORV in Mexico, the responsible authorities decided to test the bait acceptance and immunogenicity of an oral rabies vaccine bait for dogs recently licensed in the European Union (EU) under local conditions in several rural villages in Querétaro State, Mexico.

## 2. Materials and Methods

### 2.1. Study Area

**Figure 1:**
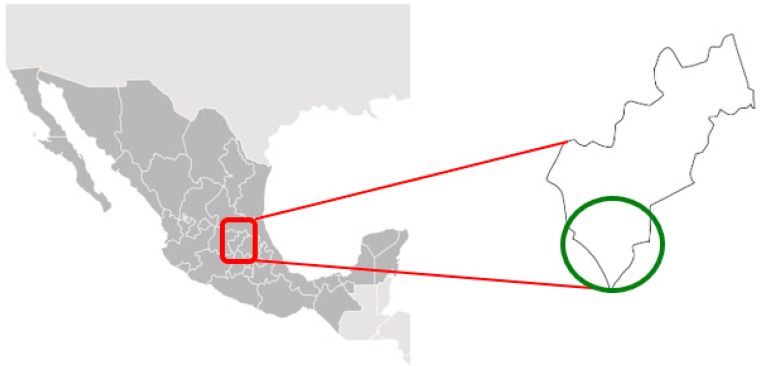
Location of study area in Querétaro State, Mexico (green circle).

Both bait acceptance and immunogenicity studies were conducted in the localities of El Vegil, Taponas, and Lagunillas, in the municipality of Huimilpan, Querétaro State, in central Mexico (Figure 1).

### 2.2. Bait Acceptance Study

To compare bait acceptance with other dog populations, a similar protocol was used as in previous studies [7,8,9,10]. The same 3 bait types as in these studies were used here: a bait made from locally available boiled (porcine) intestine segments and two manufactured baits, a fish (meal) bait and an egg (-flavored) bait. The egg bait containing a vaccine sachet with the oral rabies vaccine, SPBN GASGAS, has received marketing authorization for use in dogs in the EU and Indonesia and was therefore the focal point of interest.

Bait type to be offered to the individual dog was predetermined by randomization (www.randomizer.org, accessed on 14 February 2023). The targeted number of dogs was set at 600 animals, approximately 200 dogs per bait type.

**Figure 2:**
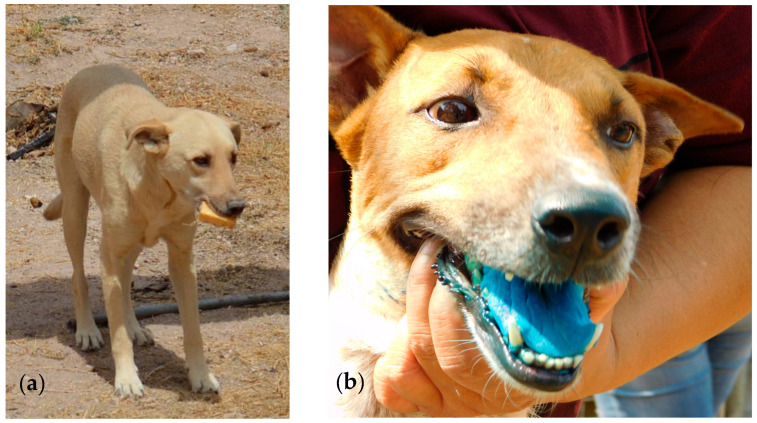
A dog offered an egg(-flavored) bait (**a**) (© R. Marquez) and the blue-coloring of the oral cavity after bait consumption (**b**) (© A. Langguth).

The sachet incorporated in the different baits contained 3.0 mL of blue-dyed water. The baits were transported to Querétaro and stored frozen and subsequently kept in cool boxes during the actual field studies. Five teams of 3–5 people were formed, and each team was allocated a specific area (village, neighborhood) on a daily basis. Prior to the study, staff were trained how to approach a dog and subsequently offer the animal a bait. The training also included data recording. Every day, group composition (partially) changed by switching staff members from one team to the other. During systematic coverage of the area, every dog encountered was offered a single bait. If the dog had an owner that could be identified, oral consent was obtained before the bait was offered to the dog. The observations were recorded on a special form for each individual dog, including detailed information on bait handling, like whether the dog was interested (direct active contact with the bait, like sniffing or licking) or not, and bait consumption (including amount and duration). Furthermore, if possible, it was reported if the sachet was perforated or not and if it was discarded or swallowed. Finally, an evaluation of the vaccination effort was made (successfully vaccinated or not) based on the staining (blue) of the oral cavity due to the release of the sachet contents [21] (Figure 2). If possible, general information on the dog was collected, including if the dog was free-roaming or restricted, owned or not, alone (solitary) or with other dogs (multiple), size, sex, and age category.

### 2.3. Immunogenicity Study

Regarding the immunogenicity study, here also a similar protocol as in previous studies was used [7,12,13]. Dogs included in the study should not have received rabies vaccination previously and should be clinically healthy. Hence, a blood sample (B0) was collected between 5 and 7 days prior to vaccination to confirm the absence of antibodies against rabies, except for 15 dogs that were sampled on the day of vaccination. Dogs should be at least 3 months of age to avoid possible interference with maternally derived immunity. Study animals were housed and fed by their owners as usual, and no special treatment was provided for the dogs during the study.

The animals were divided into three treatment groups, just as in the previous studies [7,12,13]. The dogs were to be vaccinated by offering an egg-flavored vaccine bait, by direct oral administration (d.o.a.) of the oral vaccine, or by the parenteral route using an inactivated commercially available product. First, the selected dogs were offered an egg-flavored bait containing a sachet filled with 3.0 mL of the oral rabies virus vaccine strain, SPBN GASGAS. It was targeted that approximately 25 dogs would receive the standard ‘high’ dose (10^8.1^ FFU/mL), and two smaller groups of 10 dogs each would receive 10^7.6^ FFU/mL (‘medium’—close to the Minimum Effective Dose—10^7.5^ FFU/mL [22]) or 10^7.0^ FFU/mL (‘low’). The allocation of the individual dogs to the different groups was performed ad hoc. If dogs would not accept and subsequently consume the bait, the animals were to be offered the same volume (3.0 mL) and high dose (10^8.1^ FFU/mL) of SPBN GASGAS by d.o.a., or the dogs were to be vaccinated by the parenteral route (subcutaneously [s.c.]) using 1.0 mL of an inactivated rabies vaccine (Rabiffa, Boehringer Ingelheim Promeco SA de CV, Mexico City—Mexico) as a positive control group. If the dog could easily be handled by the vaccinators, it was vaccinated by d.o.a.; otherwise, it was vaccinated parenterally. The targeted sample size was 25 dogs for each group (d.o.a. and s.c.). The oral rabies vaccine strain, SPBN GASGAS, is a replication-competent rabies virus highly attenuated through site-directed mutagenesis [23,24,25].

A second blood sample (B1) was collected from the dogs that could be relocated 34–37 days post vaccination. Blood samples were collected from the large superficial veins on the front of a front leg or on the outside of a hind leg (e.g., V. cephalica antebrachii, V. saphena), into 5 mL uncoated blood collection tubes (S-Monovette, Sarstedt, Nümbrecht, Germany) and centrifuged at 3500× *g* for 15 min. Subsequently, sera were stored at −20 °C until laboratory analysis for the presence of virus antibodies.

The serum samples were analyzed for presence of rabies virus binding antibodies (rVBA) using a commercial indirect blocking enzyme-linked immunosorbent assay (ELISA) kit (BioPro Rabies ELISA, O.K. Servis BioPro, Prague, Czech Republic) following instruction of the manufacturer. In brief, diluted serum samples were incubated on microtiter plates coated with rabies antigen. After removing the sera, all wells were incubated with a fixed amount of biotin-labeled rabies specific antibodies. Subsequently, bound antibodies were incubated with peroxidase-conjugated streptavidin followed by chromophoric detection. Intensity of color was read at 450 nm. The decrease in the intensity compared against the negative control is proportional to the amount of blocking antibodies in investigated sample. The percentage of blocking (PB) was calculated for each sample according to the manufacturer’s specifications. A percentage of blocking (PB) lower than 40% is considered as negative, a PB equal or higher than 40% is considered as positive.

### 2.4. Statistical Analysis

Univariate contingency table testing (Chi-square and Fisher’s exact test) was used for statistical analysis. For the bait acceptance study, the responses selected were “bait acceptance” and “vaccination success”, defined by (partial) consumption and the release of the contents of the sachet in the oral cavity (yes/no), respectively. The latter was made visible by the blue staining of the oral cavity, including tongue. Independent variables were bait type, date, period of the day, level of confinement, if the dog was alone (solitary) or together with other dogs (multiple), ownership status, size, gender, and age (Table 1). Weather can affect the behavior of dogs and, thus, bait acceptance and handling; therefore, the date was also taken as a variable as a surrogate for possible daily differences in weather conditions. To investigate the effect of the period of the day when baits were offered, five 2 h periods (times) were selected. Variables with *p* ≤ 0.20 were included in a multiple logistic regression model (MLR), whereby only complete data sets for the selected variables were included.

Analyzing the handling of the different bait types by the dogs, the following variables were used: proportion of bait consumed (<50%; 51–99%; 100%), duration (<10; 10–30; 31–60; >60 s), and the fate of the sachet (discarded or swallowed and perforated or not—in case the animal swallowed the sachet, this was not always possible to determine).

Statistical analyses for both bait acceptance and immunogenicity studies were carried out using GraphPad Prism v9.0 (GraphPad Prism Software Inc., San Diego, CA, USA), except for the Chi^2^ goodness-of-fit test for single variables [26].

## 3. Results

### 3.1. Bait Acceptance

A total of 618 data sets (individual dogs) were (partially) available for data analysis; an additional 17 data sets contained conflicting data and/or missing data that could not be corrected or retrieved and, thus, these were removed prior to data analysis. The proportions of the settings for each variable used for bait acceptance (see Table 1) are shown in Figure S1. Significantly more baits were offered to the dogs on the second day (*p* = 0.03), in the morning (*p* < 0.001), to owned dogs (*p* < 0.001), larger and older animals (both *p* < 0.001), and male dogs (*p* = 0.02). Significantly fewer baits were offered to solitary dogs (*p* = 0.01) (Table S1).

**Figure 3:**
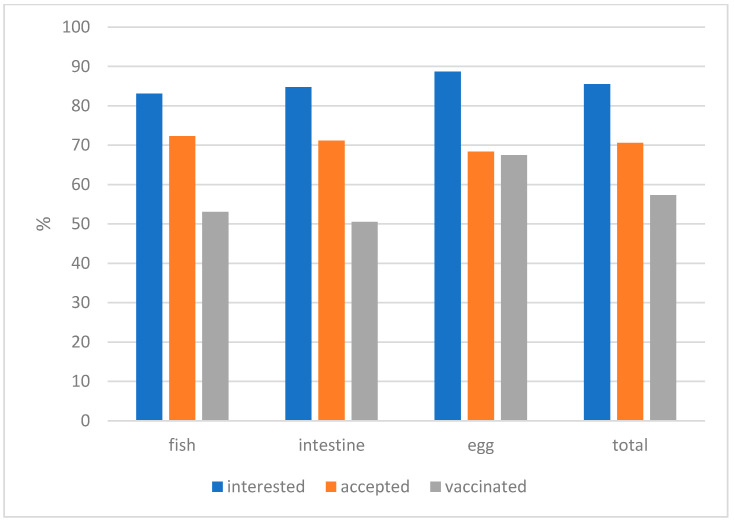
The proportion of dogs that were interested in the bait and accepted (consumed) it and the vaccination rate overall (total) and per bait type.

The raw data and the results of the statistical analysis investigating the effect of the settings for each variable on bait acceptance and vaccination rate are shown in Table 2 and Table S2. There was no significant difference in terms of interest by the dogs between the three bait types offered: intestine bait—84.3%, egg bait—87.0%, and fish bait—81.6%. However, the egg bait (68.4%) was significantly less often accepted and consumed than the other two bait types (fish—72.3%; intestine—71.2%): Chi^2^ = 6.423, df = 2, *p* = 0.04. Dogs offered an egg bait were significantly more often considered vaccinated (66.2%), irrespective of whether they were interested or not (fish—52.1%; intestine—50.3%): Chi^2^ = 13.07, df = 2, *p* = 0.002 (Figure 3).

There was also a weak significant effect (*p* = 0.045) of the period during the day the baits were offered to the dogs; during the end of the afternoon (17.00–19:00), the vaccination rate was lowest (54.29%). With increasing age, bait acceptance was also higher (*p* = 0.029). The only variable where a significant effect was observed for both bait acceptance and vaccination rate was size. Increased size of the dogs did not only result in significantly higher bait acceptance (*p* = 0.0009) but subsequently also in a higher vaccination rate (*p* = 0.041).

For bait acceptance, the following variables had a *p*-value of ≤0.20 in the univariate analysis: date, time, level of restriction, size, and age (Table S2). Unfortunately, no MLR analysis was possible as age and size were linearly dependent; in the data set, young dogs [<12 months] were always small. Only three variables (time, size, and bait type) had a *p*-value ≤ 0.20 for vaccination rate (Table S2) and were included in the MLR analysis (Table S3). The vaccination rate was higher for the egg bait and when baits were offered in the time period 15:00–16:59. Baits offered to small dogs had a lower vaccination success.

The intestine bait was significantly more often completely consumed (84%) than the egg (75%) and fish bait (69%): Chi^2^ = 10.42, df = 4, *p* = 0.0339. Chewing time was significantly shorter for the intestine bait than for the other two bait types: Chi^2^ = 88.34, df = 6, *p* < 0.0001. Forty-three (43) percent of the dogs consuming an intestine bait swallowed it within 10 s without chewing much. In contrast, 56% of the dogs needed more than 60 s to finish the egg bait. These differences in bait handling also impacted the fate of the sachets. Almost all dogs consuming an egg (85%) or fish bait (94%) discarded the sachet, while 68% of the dogs handling an intestine bait swallowed the sachet: Chi^2^ = 154.1, df = 2, *p* < 0.0001. Finally, the sachet in the egg bait (91%) was significantly more often perforated than the sachet in the fish (78%) and intestine bait (69%): Chi^2^ = 21.42, df = 2, *p* < 0.0001 (Table S4).

The amount (proportion) of bait consumed did not have a significant effect on vaccination rate: Chi^2^ = 0.3804, df = 2, *p* = 8268. However, duration of chewing time had a highly significant impact on vaccination rate: Chi^2^ = 48.86, df = 2, *p* < 0.0001. If a bait was consumed very rapidly (<10 s), vaccination success was lower, especially for the intestine bait (38%); this was less pronounced for the fish (57%) and egg bait (71%). Generally, dogs that swallowed the sachet were considered more often vaccinated (84%) than when the sachet was discarded (60%): Chi^2^ = 27.20, df = 1, *p* < 0.0001). However, there was a clear distinction between the intestine bait and the two other bait types. For the intestine bait, 93% of the dogs that swallowed the sachet were considered vaccinated. Only 49% of the dogs consuming an intestine bait and subsequently discarding the sachet were vaccinated. For the egg and fish bait, 88% of the dogs that discarded the sachet were considered vaccinated, and only 70% and 74% of the dogs that swallowed the sachet in the egg and fish bait, respectively. Only a few dogs (5/352) that perforated the sachet were not considered vaccinated (Table S5).

### 3.2. Immunogenicity

On the day of vaccination, 90 of 102 preselected dogs were vaccinated. Twelve animals (12) could not be relocated, or owners were not home and, thus, these dogs were not vaccinated. Unfortunately, for one vaccinated dog, the treatment was not recorded, and the animal was omitted from the data set. Forty-three (43) dogs were vaccinated by offering the animals a bait; 25 were offered baits containing the high dose, and 9 dogs each were given baits containing the medium and low doses. Fifteen (15) and 31 dogs were vaccinated by d.o.a. or by the s.c. route, respectively. Several dogs tested seropositive for B0 and were removed from the data set. Finally, 48 blood samples collected approximately 5 weeks post-vaccination from seronegative (B0) dogs were available: 30 from dogs offered a bait [16× high dose, 6× medium dose, and 8× low dose], 8 and 10 from dogs vaccinated by d.o.a. or s.c., respectively.

**Figure 4:**
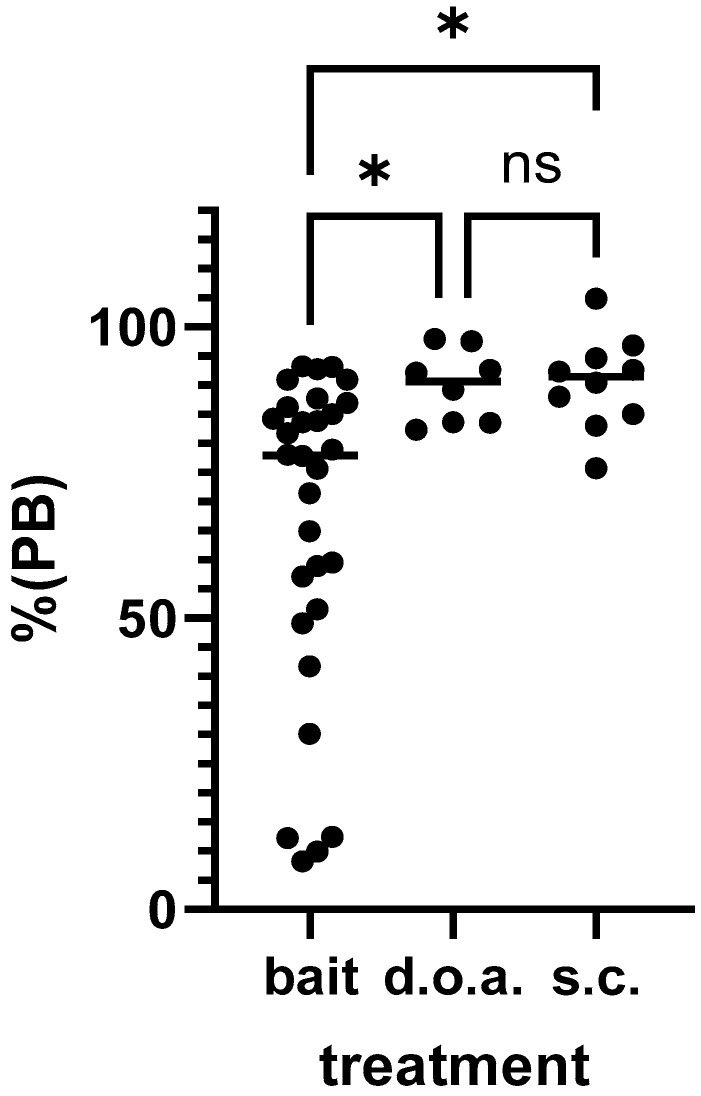
The antibody response (PB—percentage blocking) post vaccination as determined by ELISA for the individual dogs per treatment group (* —*p* < 0.05, ns—not significant).

**Figure 5:**
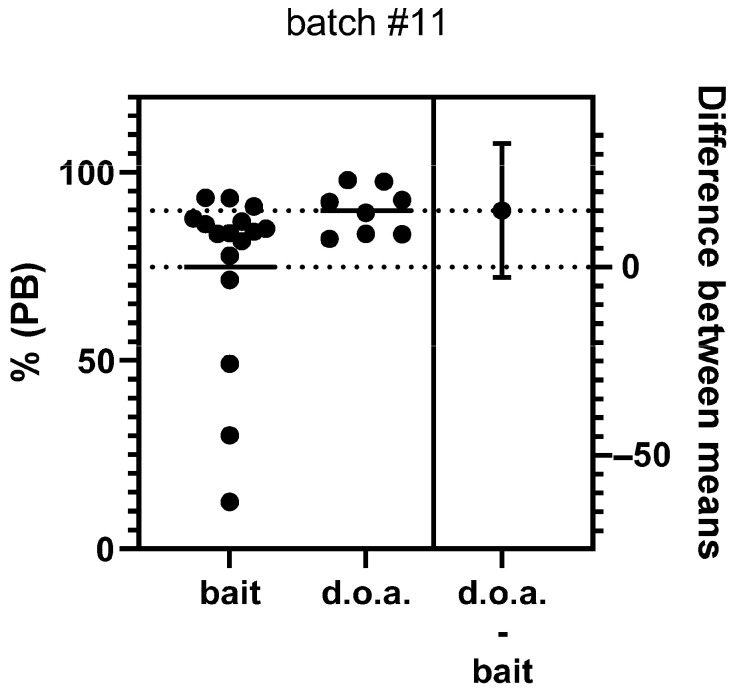
Estimation plots including the antibody response (PB—percentage blocking) post-vaccination as determined by ELISA for the individual dogs for the two treatments—bait and d.o.a. (direct oral administration)—containing the ‘high’ dose (batch#11—10^8.1^ FFU/mL) and the 95% confidence interval of the difference in the mean antibody response of both treatments.

All dogs vaccinated d.o.a. and by the parenteral route seroconverted, and 83.3% (25/30) of the dogs offered a bait. The individual PB values of the animals for the three treatment groups are shown in Figure 4. There was a significant treatment effect: one-way ANOVA, F(2,45) = 6.577, *p* = 0.031. The PB values as determined by ELISA were significantly lower in dogs offered a bait than in dogs vaccinated d.o.a. (*p* = 0.0267) or s.c. (*p* = 0.0124); Tukey’s multiple comparison test. No significant difference in PB values was observed between d.o.a. and s.c. vaccinated dogs. If we only use the data of the dogs offered a bait containing the standard ‘high’ dose, there is still a significant difference between treatment groups, but the *p*-value increased to almost non-significant levels: *p* = 0.0487 (one-way ANOVA). If we compare only the individual values for the dogs receiving the same material (high dose) by bait or d.o.a., there is no significant difference: unpaired t-test, t = 1.71, df = 22, *p* = 0.0938 (Figure 5).

**Figure 6:**
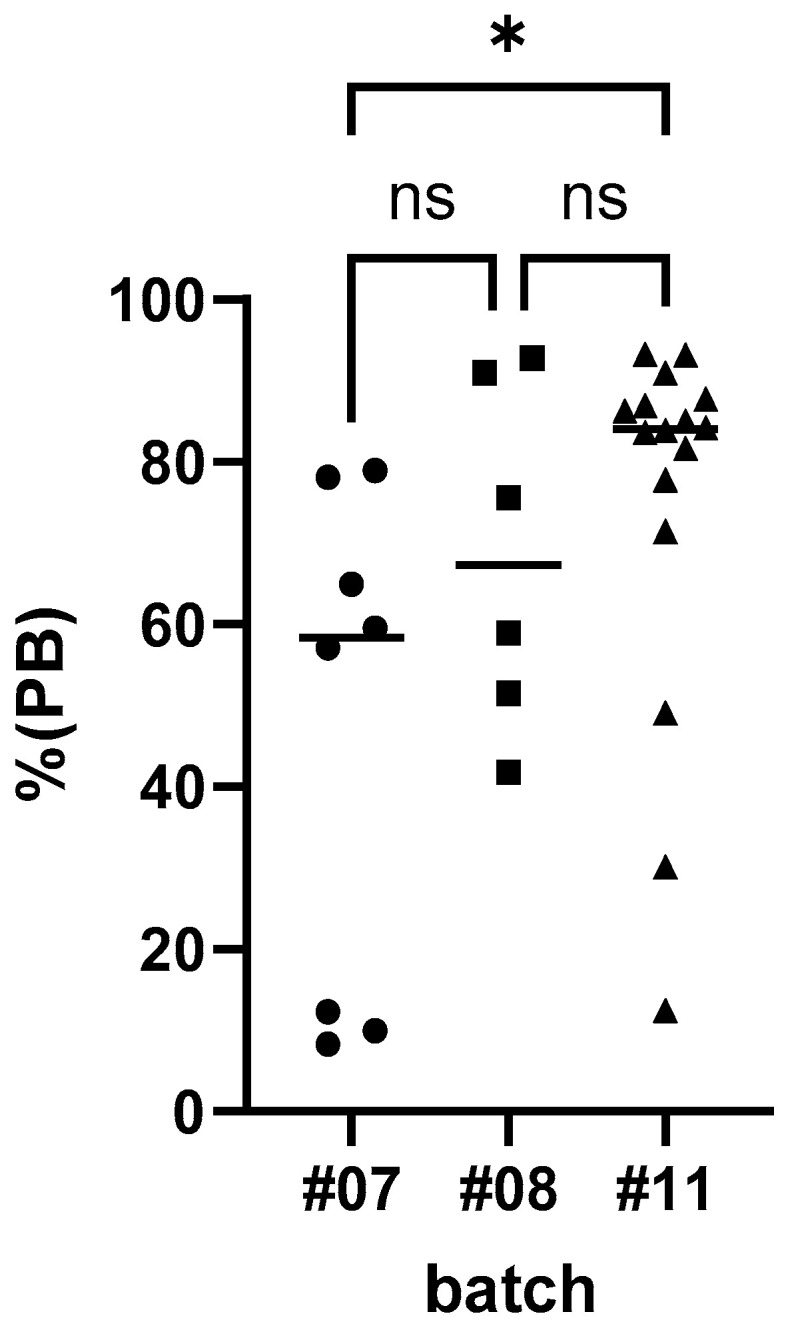
The antibody response (PB—percentage blocking) post-vaccination as determined by ELISA for the individual dogs offered baits containing the high (batch#11—10^8.1^ FFU/mL), medium (batch#8—10^7.6^ FFU/mL) and low dose (batch#7—10^7.0^ FFU/mL) (* —*p* < 0.05, ns—not significant).

Finally, the effect of the different doses was analyzed. The seroconversion rates for the high, medium, and low doses were 87.5% (14/16), 100% (6/6), and 62.5% (5/8), respectively. No significant difference in seroconversion rate between high and low doses was found: *p* = 0.2885 (Fisher’s exact test). There was a significant effect observed in the individual post-vaccination antibody levels between the different groups: one-way ANOVA, F(2,22) = 3.490, *p* = 0.0449. The PB values as determined by ELISA were significantly higher in dogs offered the high-dose bait than in dogs vaccinated with the low dose (*p* = 0.0363): Tukey’s multiple comparison test. No significant difference in PB-values was observed between high vs. medium and medium vs. low (Figure 6).

## 4. Discussion

Mexico is globally an exemplary country for how dog-mediated rabies can be locally eliminated using a One Health approach. However, in order to sustainably maintain the dog-mediated rabies-free status, innovative new strategies need to be developed and implemented. One possible complementary tool is ORV of dogs targeting free-roaming dogs that cannot easily be handled [27]. Previous studies on ORV in Mexico using another oral rabies vaccine bait had demonstrated it’s feasibility [28]. The concept of ORV is composed of the following three components: (1) a safe and efficacious vaccine, (2) a bait that is not only highly attractive for dogs but also optimizes release of the vaccine in the oral cavity, and (3) a bait distribution system that maximizes bait availability to the targeted dog population while minimizing potential exposure to non-target animals [29]. The present study in Mexico focuses on these first two components.

The bait acceptance in this study fluctuated between 68.4 (egg bait) and 72.3% (fish bait), depending on bait type. This acceptance rate is lower than observed in a previous study in Mexico, where 97% of owned dogs reportedly accepted the bait [28]. However, much lower bait acceptance rates were observed for four different bait types during another field study in Mexico. Here, bait acceptance was between 10% and 69% [30]. Here, the highest acceptance rate was obtained with a bait containing fish oil as an attractant. Also, in the present study, the highest bait acceptance of the three baits tested was the fish bait (72.3%). Interestingly, during acceptance studies using the same three bait types, the fish bait was always the bait type with the lowest acceptance rate compared to the other two bait types, except in Navajo Nation (Table S6) [7,8,9,10]. The results of an earlier bait acceptance study with different bait types in Navajo Nation also showed that a fish-flavored coated sachet had the highest bait consumption among 4 different bait types examined [31]. Hence, local preferences differ and can have an effect on bait acceptance. Often familiarity with taste and texture plays an important role. Hence, a bait made from locally available material seems to be the most promising candidate. Unfortunately, a vaccine bait is a pharmaceutical product and is therefore subject to strict regulatory requirements. Introducing changes to the final product, including the bait matrix, could require additional efficacy studies (with a challenge infection). Therefore, a universal, well-accepted bait with a standardized composition is preferable, even if locally better-accepted baits are available.

Although the egg bait was accepted slightly less often than the other two baits, dogs offered the egg bait were considered significantly more often successfully vaccinated. The difference between the acceptance and vaccination rate for the egg bait was relatively small compared to the other two baits, indicating optimal release of the sachet contents when the animal consumes the bait. The same observation was made during previous studies; also, here the egg bait was not always the most preferred bait but consistently had the highest vaccination rate [7,8,9,10]. The fish bait matrix is identical to the baits used for ORV of foxes and raccoon dogs in Europe and consists, among others, of fish meal and vegetable fatty substances [32]. As this bait easily falls apart when chewed on by dogs, the bait matrix and sachet are separated, and consequently the dogs do not perforate the sachet. The intestine is often swallowed without chewing and, thus, also under these circumstances, the sachet will not be punctured. In both cases, the baits were consumed but did not result in a successful vaccination attempt. The dogs are most of the time not familiar with the texture of the egg bait; consequently, there is indecision if the bait is ‘edible’ or not. This results in prolonged chewing time and a high perforation rate of the sachet compared to the other bait types. During the present study, it was observed that on several occasions a dog would not consume the egg bait when offered, but the animal would perforate the sachet before dropping the bait, and the contents (blue-dyed water) were subsequently released in the oral cavity. Besides the higher bait acceptance rate of the fish meal bait in Mexico compared to most of the other studies, the significant effect of the period of the day on bait acceptance also differed from most other studies. In Mexico, bait acceptance and vaccination rate were higher between 15:00 and 17:00 than during the other periods studied. In other studies, in Thailand, Indonesia, and India, no preferred period of the day for bait acceptance was observed [9,10,15].

In the present study, a blocking ELISA was used to evaluate the immune response. Previous immunogenicity and efficacy studies in orally vaccinated animals, including dogs, had shown that the outcome of this ELISA was better suited as an indicator of protective immunity than a virus neutralization assay like RFFIT and for detecting long-term immunity [22,33,34,35].

The serology results confirmed the general observation that the seroconversion rate in dogs offered a bait is slightly lower than when the vaccine is given by direct oral administration or by parenteral vaccination using an inactivated vaccine (Table S7). Also, the antibody levels in the individual dogs offered a vaccine bait showed a larger variation than in the animals from the other two groups. These observations are explained by the way the individual dogs handle the baits offered. Some dogs swallow the bait without any chewing. Consequently, the sachet is not perforated, and the vaccine is not released in the oral cavity, a prerequisite for the induction of an immune response, as the vaccine virus needs to enter the host via the mucous membrane in the oral cavity, especially the palatine tonsils [36]. In case the dog perforates the sachet, some vaccine will be swallowed immediately or spilled on the ground. Hence, the available amount of vaccine for uptake via the oral mucous membrane differs among the dogs, and consequently some dogs that accept and consume a bait will subsequently not be vaccinated successfully. For example, 5 of 352 dogs that had discarded the perforated sachet were not considered vaccinated during the bait acceptance study. The sometimes suboptimal vaccine delivery in the oral cavity is the reason that a relatively high dose (FFU/mL) needs to be administered. As mentioned before, the discrepancy between acceptance and vaccination rate is relatively small for the egg bait. During the initial immunogenicity studies in Thailand, the boiled intestine bait containing a different vaccine-filled PVC capsule was used [20]. In subsequent immunogenicity and challenge studies, the present egg bait containing a vaccine-filled soft blister was used [7,12,13,22]. Although this bait is more efficient than the intestine bait in releasing the contents of the sachet in the oral cavity [22], the same vaccine dose (Foci Forming Units [FFU] per mL) was used in these field studies. Hence, it was decided to test if the vaccine dose could be lowered in the egg-flavored bait during the study in Mexico. Three of the eight dogs that received a bait containing the low-titred vaccine material did not seroconvert. Although no significant difference was found in the seroconversion rate between dogs receiving baits containing the high and low dose, the sample size was too small for conclusive evidence that the low dose would also suffice, especially considering that a significant difference in the individual antibody level in dogs receiving the high and low doses was found (Figure 6).

It is extremely important to point out that oral vaccination is not targeted at the individual animal but is intended to increase herd immunity, especially of the free-roaming dogs [29]. In Thailand, it was shown that 79.0% of the targeted ownerless free-roaming dog population could be offered a bait, and subsequently 83.0% of these dogs were considered successfully vaccinated, resulting in an overall vaccination coverage of 65.6% of these dogs inaccessible for parenteral vaccination [17].

This study showed that this licensed vaccine bait is well accepted by local dogs in Mexico and can achieve a high seroconversion rate. Future studies in Mexico should focus on the third component of ORV: the bait distribution system. How and where to distribute the baits to the targeted subpopulation of dogs in the most (cost-) effective way, contributing to the dog-mediated rabies-free status of the country.

## 5. Conclusions

- Rabies remains a public health threat in Mexico as the rabies virus continues to circulate among wild animals and can be transmitted to humans and domestic animals like dogs.
- The egg bait was not the most preferred bait by dogs in Mexico but achieved the highest vaccination rate of all three bait types offered.
- Although vaccination of dogs offered a vaccine bait that was slightly less effective (seroconversion rate) than parenteral vaccination under field conditions in Mexico, ORV can increase the overall vaccination coverage by reaching a significant part of the dog population that cannot readily be handled for vaccination.

This article is a revised and expanded version of a presentation entitled “Feasibility of oral rabies vaccination of dogs in Mexico: bait acceptance and immunogenicity studies”, which was presented at the International Conference Rabies in the Americas, 16–19 October 2023, Bogota–Columbia [37].

## Figures and Tables

**Table 1:** Parameters (variables) and settings used during the bait acceptance study.

Variable	Settings
date	26 April; 27 April; 28 April
time (hh:mm)	09:00–10:59; 11:00–12:59; 13:00–14:59; 15:00–16:59; 17:00–19:00
level of restriction	restricted, free-roaming
social status	solitary; multiple
ownership	owned, ownerless, community
size	small; medium; large
sex	female; male
age	<12 months; ≥12 months
bait type	egg, intestine, fish meal

**Table 2:** Number and proportion of dogs consuming the bait (acceptance) and considered vaccinated (vaccination) during the bait acceptance study for each setting and variable (n/N is n—number of dogs that accepted the bait or number of dogs considered to be vaccinated and N—total number of dogs offered a bait).

Variable	Settings				
date	26 April	27 April	28 April		
acceptance	107/170 (64.85%)	174/240 (73.11%)	151/208 (73.30%)		
vaccination	90/170 (56.25%)	132/240 (58.41%)	127/208 (62.25%)		
time	9:00–10:59	11:00–12:59	13:00–14:59	15:00–16:59	17:00–18:59
acceptance	83/126 (66.40%)	199/277 (73.70%)	71/104 (68.93%)	58/73 (79.45%)	19/35 (54.29%)
vaccination	67/126 (54.92%)	166/277 (61.94%)	55/104 (56.70%)	45/73 (67.16%)	15/35 (45.45%)
restriction	restricted	free-roaming			
acceptance	229/313 (73.40%)	187/283 (67.51%)			
vaccination	179/313 (59.67%)	159/283 (58.89)			
social status	solitary	multiple			
acceptance	246/343 (69.82%)	183/256 (72.33)			
vaccination	194/343 (59.33%)	159/283 (59.35%)			
ownership	owned	ownerless	community		
acceptance	252/349 (72.62%)	104/150 (71.23%)	16/27 (61.54%)		
vaccination	201/349 (60.18%)	82/150 (56.94%)	16/27 (61.54%)		
size	small	medium	large		
acceptance	67/118 (57.26%)	146/202 (73.00%)	206/277 (75.74%)		
vaccination	56/118 (50.45%)	116/202 (59.18%)	170/277 (64.39)		
sex	female	male			
acceptance	179/246 (73.36%)	229/332 (70.03%)			
vaccination	143/246 (60.59%)	186/332 (58.68%)			
age	<12 months	≥12 months			
acceptance	45/74 (60.81)	320/435 (74.42%)			
vaccination	43/71 (58.11%)	247/435 (59.66%)			
bait type	egg	intestine	fish meal		
acceptance	145/216 (69.58%)	131/185 (70.81%)	154/217 (70.97%)		
vaccination	143/216 (66.20%)	93/185 (50.27%)	113/217 (52.07%)		

## Data Availability

The original contributions presented in this study are included in the article/Supplementary Material. Further inquiries can be directed to the corresponding author.

## Supplementary Materials

The following supporting information can be downloaded at: https://www.mdpi.com/article/10.3390/tropicalmed10090244/s1, Figure S1: The observed proportions during the bait acceptance study of the different settings for each variable included as listed in Table 1 (for example: date—27% of the baits were offered on 26 April); Table S1: The statistical analysis (Chi^2^ goodness-of-fit test) for the proportions of the settings of each variable for the number of baits offered to the dogs during the bait acceptance study; Table S2: The statistical analysis (Chi^2^ goodness-of-fit test) for the proportions of the settings of each variable for the number of dogs accepting (consuming) and considered vaccinated during the bait acceptance study; Table S3: The Odds Ratio and the Confidence Interval of the Multiple Logistic Regression Model; Table S4: Statistical analysis of bait handling by dogs for each bait type and the proportions of each setting for the variables investigated; Table S5: Vaccination rate for each bait type and setting for the variables investigated for bait handling; Table S6: Acceptance rate of the egg-, fish- and intestine bait in the different countries; Table S7: Seroconversion rate in dogs offered a vaccine bait (egg-bait) containing SPBN GASGAS, offered SPBN GASGAS by direct oral administration (d.o.a.) or vaccinated by the parenteral route using an inactivated vaccine in the different studies.
