# Supplementary material for: Feasibility of Oral Rabies Vaccination of Dogs in Mexico
Source: Trop Med Infect Dis. 2025 Aug 28;10(9):244. doi: 10.3390/tropicalmed10090244 (PMC12474024; doi:10.3390/tropicalmed10090244)
Supplement: Supplementary file 1 [file tropicalmed-10-00244-s001.zip › tropicalmed-3768029-supplementary.pdf]

Tropical Medicine and Infectious Disease

MDPI

Supplementary Materials

Table S1. The statistical analysis (Chi² goodness-of-fit test) for the proportions of the settings (see table 1) of each variable for the number of baits offered to the dogs during the bait acceptance study (data entries 'other/unknown' were not removed for statistical analysis); Chi² - test value, df = degrees of freedom, p = p-value whereby $p < 0.05$ is considered significant).

Variable	Chi²	df	*p*
date	11.92	2	0.003
time (hh:mm)	86.22	4	<0.001
level of restriction	1.18	1	0.28
social status	6.78	1	0.01
ownership	174.14	2	<0.001
size	57.05	2	<0.001
sex	5.62	1	0.02
age	356.32	2	<0.001
bait type	3.21	2	0.20

Table S2. The statistical analysis (Chi² goodness-of-fit test) for the proportions of the settings (see table 1) of each variable for the number of dogs accepting (consuming) and considered vaccinated during the bait acceptance study (data entries 'other/unknown' were not removed for statistical analysis); Chi² - test value, df = degrees of freedom, p = p-value whereby $p < 0.05$ is considered significant).

	Bait Acceptance			Vaccination Rate		
Variable	Chi²	df	p	Chi²	df	p
date	4.070	2	0.13	1.422	2	0.49
time (hh:mm)	9.730	4	0.045	6.353	4	0.17
level of restriction	2.452	1	0.117	0.036	2	0.850
social status	0.442	1	0.665	0.00003	1	0.99
ownership	1.483	2	0.476	0.586	2	0.746
size	14.1000	2	0.0009	6.388	2	0.041
sex	0.760	1	0.383	0.207	1	0.650
age	4.770	1	0.029	0.015	1	0.90
bait type	2.802	2	0.25	18.96	2	<0.0001

Table S3. The Odds Ratio and the Confidence Interval of the Multiple Logistic Regression Model, as a reference, time period 9:00 – 11:00, fish meal bait, size –large were selected.

Variable	Setting	Estimate	95% CI (Profile Likelihood)
	Intercept	1.293	0.8150 to 2.061
time	11:00 – 12:59	1.199	0.7590 to 1.888
	13:00 – 14:59	0.9934	0.5637 to 1.753
	15:00 – 16:59	1.994	1.032 to 3.952
	17:00 – 18:59	0.6428	0.2869 to 1.423
size	medium	0.7453	0.5007 to 1.109
	small	0.5026	0.3137 to 0.8018
bait	egg	2.108	1.380 to 3.245
	intestine	0.9515	0.6222 to 1.455

Table S4. Statistical analysis of bait handling by dogs for each bait type and the proportions of each setting for the variables investigated. (data entries 'other/unknown' were not removed for statistical analysis); Chi² - test value, df= degrees of freedom, p = p-value whereby $p < 0.05$ is considered significant).

Variable	Settings	Egg	Fish	Intestine	Chi²	df	*p*
Consumption (%)							
	≤50	13.85	11.03	7.38	10.42	4	0.0339
	51-99	11.54	19.85	9.02			
	100	74.62	69.12	83.61			
Duration consumption (sec)							
	<10	10.64	5.44	43.28	88.34	6	<0.0001
	10-30	12.06	19.05	15.67			
	31-60	21.28	33.33	17.16			
	>60	56.03	42.18	23.88			
Sachet I							
	swallowed	14.79	5.84	68.22	154.1	2	<0.0001
	discarded	85.21	94.16	31.78			
Sachet II							
	perforated	90.51	77.78	68.89	21.42	2	<0.0001
	not-perforated	9.49	22.22	31.11			

Table S5. Vaccination rate for each bait type and setting for the variables investigated for bait handling. Statistical analysis was only performed for the pooled results of all bait types as the requirements of the Chi²-test were not met for the individual bait types. (data entries 'other/unknown' were not removed for statistical analysis); Chi² - test value, df = degrees of freedom, p = p-value whereby $p < 0.05$ is considered significant).

Variable	Settings	Egg	Fish	Intestine	Overall	Chi²	df	*p*
Consumption (%)								
	≤50	94.44	46.67	87.50	75.61	0.3804	2	0.8268
	51-99	80.00	76.00	90.91	80.39			
	100	91.40	77.42	61.70	76.79			
Duration consumption (sec)								
	<10	71.43	57.14	38.46	46.58	48.86	2	<0.0001
	10-30	82.35	78.57	80.95	80.30			
	31-60	96.55	70.21	95.65	83.84			
	>60	93.42	75.81	86.67	85.71			
Sachet I								
	swallowed	70.00	74.13	92.50	84.16	27.20	1	<0.0001
	discarded	87.50	87.50	48.91	60.00			
Sachet II								
	perforated	99.28	96.58	100	98.58	not applicable		
	not perforated	0	0	0	0			

Table S6. Acceptance rate of the egg-, fish– and intestine bait in the different countries (n—number of baits offered that were accepted, N—number of baits offered, 95%CI – 95% confidence interval).

Country		Egg- Bait	Fish- Bait	Intestine- Bait	Reference
Navajo Nation	n/N	292/370	73/90	79/86	Bender et al., 2017 [8]
	%	78.92	81.11	91.86	
	95%CI	75.13–82.36	73.02–87.59	85.26–96.12	
Thailand	n/N	141/192	86/206	137/192	Kasemsuwan et al., 2018 [10]
	%	73.44	41.75	71.35	
	95%CI	67.68 – 78.64	35.97–47.70	65.60–76.71	
Indonesia	n/N	254/317	204/348	316/327	Husein et al., 2023 [9]
	%	80.13	58.62	96.64	

	95%CI	76.08 – 83.75	54.09–63.04	94.49–98.10	
Morocco	n/N	37/47	23/50	41/42	Aboulfidaa et al., 2024 [7]
	%	78.72	46.00	97.62	
	95%CI	66.59 – 87.98	33.85–58.52	89.20–99.88	
Mexico	n/N	145/212	154/213	131/184	present study
	%	68.40	72.30	71.20	
	95%CI	62.74–73.67	66.81–77.32	65.20–76.67	

Table S7. Seroconversion rate in dogs offered a vaccine bait (egg-bait) containing SPBN GASGAS, offered SPBN GASGAS by direct oral administration (d.o.a.) or vaccinated by the parenteral route using an inactivated vaccine in the different studies. The seroconversion rate is based on the antibody response assessed by ELISA approx. one month post vaccination except for Morocco where no sample prior to 450 days post vaccination was available (n—number of baits offered that were accepted, N—number of baits offered, 95%CI – 95% confidence interval).

Country		Bait	d.o.a.	Parenteral	Reference
Namibia	n/N	20/26	6/7	2/2	Molini et al., 2021 [21]
	%	76.92	85.71	100	
	95%CI	59.46–89.44	47.93–99.27	22.36–100	
Thailand	n/N	15/15	10/10	10/10	Leelahapong-sathon et al., 2020 [20]
	%	100	100	100	
	95%CI	81.90–100	74.11–100	74.11–100	
Indonesia	n/N	40/45	16/17	20/22	Saputra et al., 2023 [12]
	%	88.88	94.11	90.91	
	95%CI	78.05–95.92	74.99–99.70	74.05–98.36	
Morocco	n/N	16/19	5/5	4/4	Aboulfidaa et al., 2024 [7]
	%	84.21	100	100	
	95%CI	64.06–95.55	54.93–100	47.29–100	
Mexico	n/N	25/30	8/8	10/10	present study
	%	83.33	100	100	
	95%CI	68.10–93.19	68.77–100	74.11–100	

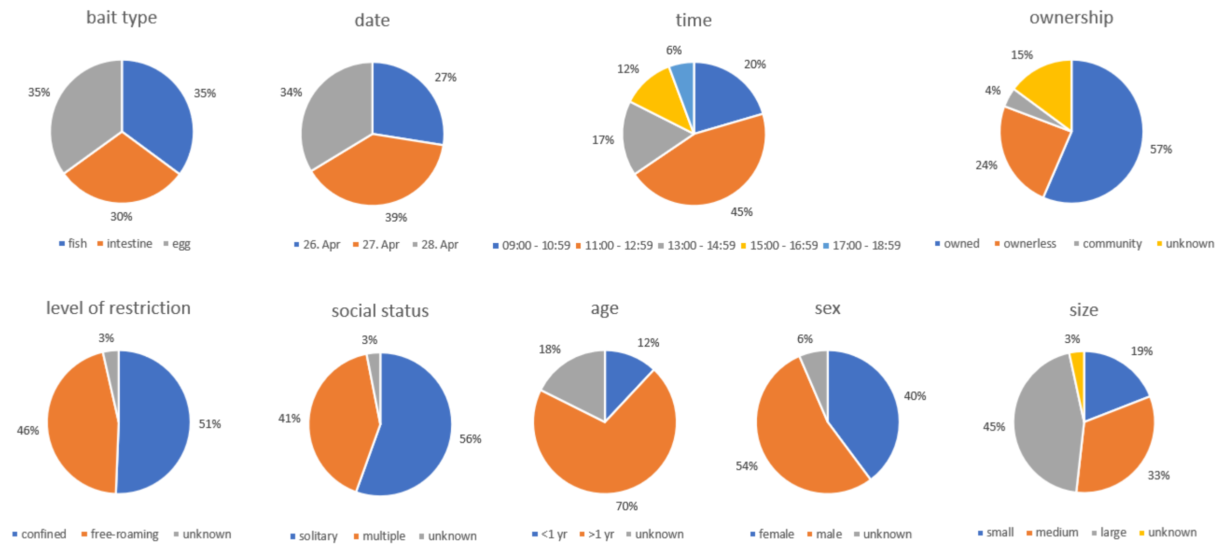

Figure S1. The observed proportions during the bait acceptance study of the different settings for each variable included as listed in Table 1 (for example: date – 27% of the baits were offered on April 26th).

Disclaimer/Publisher's Note: The statements, opinions and data contained in all publications are solely those of the individual author(s) and contributor(s) and not of MDPI and/or the editor(s). MDPI and/or the editor(s) disclaim responsibility for any injury to people or property resulting from any ideas, methods, instructions or products referred to in the content.
